# Characteristics of Myelodysplastic Syndrome with Coagulation Abnormalities and Tailored Diagnosis and Treatment

**DOI:** 10.3390/jpm15090429

**Published:** 2025-09-05

**Authors:** Osamu Imataki, Makiko Uemura, Akira Kitanaka

**Affiliations:** 1 Department of Laboratory Medicine, Kawasaki Medical School, Kurashiki 701-0192, Japan; kitanaka@med.kawasaki-m.ac.jp; 2 Department of Laboratory Medicine, Kawasaki Medical School, General Medical Center, Okayama 700-8505, Japan; 3 Division of Hematology, Department of Internal Medicine, Faculty of Medicine, Kagawa University, Miki-town, Kagawa 761-0701, Japan; uemura.makiko@kagawa-u.ac.jp

**Keywords:** myelodysplastic syndrome, coagulopathy, coagulation, disseminated intravascular coagulation, tumor lysis syndrome, hemophagocytic syndrome/hemophagocytic lymphohistiocytosis, paraneoplastic syndrome, tailored medicine, personalized medicine

## Abstract

At onset, myelodysplastic syndrome (MDS) may be complicated by coagulation and fibrinolytic abnormalities, such as disseminated intravascular coagulation (DIC), tumor lysis syndrome (TLS), infection, thromboembolism, hemophagocytic syndrome/hemophagocytic lymphohistiocytosis (HPS/HLH), hemorrhage, and hematoma formation. In these cases, the cause may be secondary. On the other hand, it is known that platelet clotting dysfunction and fibrinolysis abnormalities are seen in the background of MDS, and primary fibrinolysis abnormalities may be complicated by adverse events associated with paraneoplastic syndrome (PNS). Coagulation fibrinolysis, as a PNS associated with MDS, is known to take the pattern of either consumptive coagulation abnormality or fibrinolytic coagulation abnormality. One mechanism of coagulation and fibrinolytic abnormalities has been shown to be the immunophenotypical pathway, and aberrant cytokine production is also associated with coagulopathy in MDS. We focused on how to differentiate an MDS-associated bleeding tendency resulting from either secondary or primary causes. In order to make this differentiation, we proposed a useful flowchart for the differentiation of solidified fibrinolysis seen at the initial MDS diagnosis. Additionally, we compared and summarized the molecular pathways of the secondary and primary causes of coagulopathy. Addressing coagulation and fibrinolytic abnormalities in MDS is required to differentiate the complexity and heterogeneity of bleeding and coagulation abnormalities. This review highlights the need to distinguish between the primary (disease-intrinsic) and secondary (reactive or complication-related) causes of coagulopathy. By proposing a diagnostic flowchart tailored to evaluate these causes at initial diagnosis, this study supports individualized risk stratification and management strategies. By comparing the molecular pathways of the two causes of coagulopathy, we provide a clinical discussion of the underlying pathologies. This aligns with the principles of personalized medicine by ensuring that treatment decisions (e.g., supportive care, anticoagulation, and antifibrinolytics) are based on the patient’s specific pathophysiological profile, rather than a one-size-fits-all approach.

## 1. Introduction

Myelodysplastic syndrome (MDS) is a diverse bone marrow function disorder caused by an acquired genetic abnormality, presenting progressive cytopenia refractory to nutritional or growth factor treatment [1]. The main hematologic finding in MDS is anemia, but it sometimes occurs alongside neutropenia or thrombocytopenia [2]. MDS primarily affects the elderly (most patients are older than age 65), but it can also occur in a small number of younger patients.

**Figure 1:**
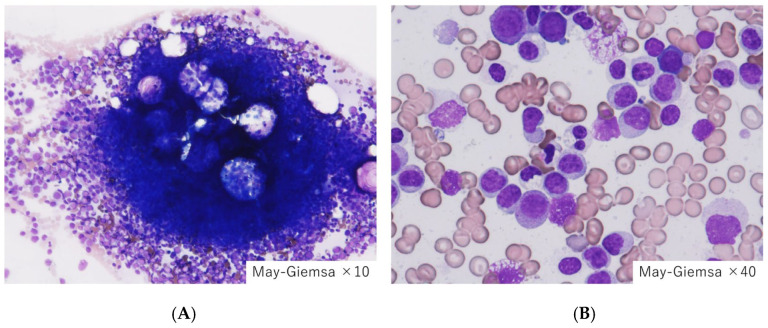
Bone marrow findings in the reference case. (**A**) Hypercellular marrow, (**B**) Increased blasts. Our indexed patient with myelodysplastic syndrome with multilineage dysplasia (MDS-MLD) indicated hypercellular marrow, where the myeloid blasts count exceeded 10%. Disseminated intravascular coagulation (DIC) met the diagnostic criteria; however, it was not accompanied by tumor lysis syndrome or infection.

Some MDS patients may develop a paraneoplastic consumptive coagulopathy or fibrinolytic process. Platelet dysfunction associated with MDS can contribute to bleeding tendencies. MDS-related platelet dysfunction and coagulopathy may lead to bleeding complications in affected individuals. Our study focusses on the following narrative case of MDS-complicated severe coagulopathy as a reference case:

A 51-year-old male was referred to the hematology outpatient clinic with a chief complaint of fever. Blood tests revealed leukopenia and coagulation–fibrinolysis abnormalities. The diagnosis was febrile neutropenia. The bone marrow examination showed hyperplastic myeloid cells, with no significant increase in blast cells (0.1%). Quantitative dysplastic changes were observed in both granulocytic and erythroid lineages, leading to a diagnosis of myelodysplastic syndrome with multilineage dysplasia (MDS-MLD) (Figure 1). No phagocytosis was seen in the bone marrow. Although the initial platelet count was mildly decreased (128,000/μL), criteria for disseminated intravascular coagulation (DIC) were met [3], indicating hyperfibrinolysis-type DIC. The patient had no history of liver cirrhosis, and hepatitis B virus antigen and hepatitis C virus antibodies were negative. There was no increase in blast cells or elevated lactate dehydrogenase (LDH). Tumor lysis was ruled out, and CT scans showed no splenomegaly or thromboembolic findings.

As indicated in this case, we often encounter coagulopathy cases in which the underlying disease should be distinguished. This is because primary coagulopathy in MDS is uncommon. Here, we considered the primary abnormality underlying coagulation and fibrinolysis to be paraneoplastic syndrome (PNS) associated with MDS. In what follows, we offer a means of differentiating primary and secondary coagulation and fibrinolysis disorders. The goal of this review is to support a medical approach that tailors treatment and prevention strategies to the unique pathogenesis of each underlying disease.

## 2. Pathogenic Mechanism

Inherited thrombocytopenia with platelet dysfunction is known as a genetic disease [4]. In this review, we do not mention this type of coagulopathy-exhibiting inherited disease.

The incidence of thrombocytopenia in MDS is 67% before treatment [2]. In a review article published in 2018, platelet dysfunction associated with MDS was comprehensively described [5]. The molecular mechanisms and cytokine milieu involved in platelet dysfunction were reviewed. From the perspective of laboratory medicine, platelet function was tested using various coagulation factors, such as arachidonic acid (54%), ADP (46%), collagen (43%), and ristocetin (22%) [6]. These data indicated a roughly 50% decrease in platelet coagulation properties. Despite the relatively high incidence of platelet dysfunction, bleeding in MDS patients is uncommon.

In patients with MDS, platelets showed reduced activation capacity and more apoptosis signs than controls [7]. Some patients with MDS present with bleeding symptoms due to platelet dysfunction, even when platelet counts are within a normal range. Platelet aggregation dysfunction is commonly present in MDS. One key mechanism involves impaired glycoprotein VI (GPVI) signaling in platelets. Despite normal GPVI surface expression, some MDS patients show defective aggregation to GPVI ligands such as collagen and collagen-related peptide. In a small laboratory study, platelet aggregation function was defective in 21 out of 26 patients (80.7%) with MDS [8]. The function was most seriously dismissed in the patients with excess blast. Although functional deficit of platelet aggregation in MDS patients did not elicit clinical hemorrhagic events, defective platelet aggregation was correlated with poor prognosis. Bleeding episodes were typically mild but recurrent. In MDS patients, aberrant phenotypes on platelets are already reported, such as CD154 and TLR4 [9] and CD61 [10] down-regulation. Additionally, associated with phenotypic changes, giant platelets are shown more frequently in MDS patients [10]. MDS patients showed reduced platelet antigen presentation compared to healthy controls, and the presence of giant platelets was associated with the disease condition [10]. In another study, altered immunophenotypic patterns were detected in MDS, which displayed decreased CD36, CD61, and/or CD42a expression and positivity for CD34 [11]. These MDS-specific immunophenotypic alterations were associated with thrombocytopenia, megakaryocytic dysplasia, and MDS disease risk [11]. These alterations mean that decreased expression of specific glycoproteins hinders platelet aggregation, and the degree of these alterations is associated with patients’ prognosis.

Based on clinical observation, coagulopathy related to thrombin formation has been shown in MDS patients [12]. Patients with MDS have deteriorated hemostasis due to decreased thrombin generation not related to thrombocytopenia [12]. This may cause a bleeding tendency in patients with MDS, especially after invasive interventions such as central venous catheter insertion. In a minor case, an acquired factor VIII inhibitor was found, causing a clinical bleeding tendency [13]. Clinical investigations revealed that platelet aggregation function is decreased due to lower expression of surface antigens such as GPIIb and GPIIIa [9] in patients with MDS [14].

In recent research, VEXAS (vacuoles, E1 enzyme, X-linked, autoinflammatory, somatic) syndrome was proposed as an autoimmune disease, exhibiting auto-inflammation presenting arthritis and vasculitis [15]. VEXAS is an acquired adult-onset auto-inflammatory disease, whose essential pathogenesis is hematological neoplasms caused by UBA1 gene alteration in hematopoietic cells [16]. UBA1 mutated cells release proinflammatory cytokines (TNF-α, IL-6, and IFN-γ) which exert varied systemic inflammatory reactions. VEXAS also induces vasculitis and associated thromboembolism; it might be an interesting theme in the elucidation of inflammation and coagulopathy in MDS disease in future.

## 3. Disseminated Intravascular Coagulation (DIC)

A combination of the keywords “MDS” and “disseminated intravascular coagulation (DIC)” yielded only 14 results in a PubMed search (on 7 July 2025). However, no fully matched articles were found. This suggests that MDS is commonly comorbid with DIC. Increased blast counts can promote the comorbidity of DIC [17], and half of the reported studies investigating DIC and AML included MDS patients (with excess blasts) as a target population [17,18]. A cohort study investigated the occurrence of thrombosis in AML and high-risk MDS. The authors claimed that the presence of DIC at diagnosis was an independent risk factor for mortality, regardless of thrombosis formation [18]. They demonstrated that the DIC score at diagnosis, rather than thrombosis, was independently associated with reduced survival. Thus, we may underestimate the morbidity of DIC at diagnosis as a prognostic factor.

## 4. Tumor Lysis Syndrome (TLS)

Similarly to the search for MDS and DIC, a PubMed search using the keywords “MDS” and “tumor lysis syndrome (TLS)” yielded sixteen results (on 7 July 2025) with only two valuable articles. These results included a case of infection-induced TLS in an MDS patient complicated by fungemia [19] and a case of progressive blastic plasmacytoid dendritic cell neoplasm arising from MDS complicated by TLS [20]. As presented in these case reports, spontaneous TLS can occur in special situations and diseases, although it is more common in oncology [21,22,23]. Currently, more than 120 case reports are indexed in the PubMed collection (as of 15 July 2024). Although spontaneous TLS is common clinical observation in clinical oncology [23], there are no reports specifically for MDS. However, given that a high tumor cell proliferation rate and size are associated with the risk and incidence of TLS [24], it is not inconceivable for this event to occur in MDS patients as well.

A single case report introduced a case of low-risk MDS with TLS without chemotherapy or immunotherapy [25]. In this case, the primary manifestation was acute kidney injury caused by hyperuricemia and TLS, and the patient was subsequently diagnosed with MDS about one year later. This indicates that TLS can precede the onset of clinical or symptomatic MDS. Acute kidney injury caused by hyperuricemia and TLS was the primary manifestation in this case. This case was educational for hematologists because cytolysis can present as a critical condition. We may observe trigger diseases such as infection and surgical intervention which can induce inflammation. Such events can easily disturb the cytokine milieu.

## 5. Infection

Infection is a common complication in MDS [26], because neutrophils in MDS patients have impaired function [27,28]. In vitro studies have reported defective neutrophil function [27], making MDS patients clinically vulnerable to both bacterial and fungal infection [28]. The leading cause of death in MDS is disease progression [29], with infection being the second leading cause, estimated to cause 17.8% of deaths [29]. Therefore, infection management is crucial among all supportive care measures to ensure the survival of MDS patients [30].

## 6. Thromboembolism

Thromboembolism associated with MDS is partially related to some key drugs used to treat MDS, such as lenalidomide [31], growth factors (darbepoetin alpha) [32,33], and thalidomide [32]. For darbepoetin, the increased incidence of thromboembolism is still controversial [34]. The association between venous thrombosis and lenalidomide, in particular, has a major significant impact [35]. Additionally, there is scientific interest in the contribution of MDS itself to the increased clinical incidence of thromboembolism.

MDS can be associated with an increased risk of thromboembolism, including deep vein thrombosis or pulmonary embolism. The exact mechanism behind this increased risk is not fully understood, but several factors contribute to it. We propose summarizing contributing factors as two sets of ABC.

The first ABC is MDS-based risk factors:

(1) Abnormal blood cells: MDS involves dysfunctional blood cell production, including abnormal red blood cells, white blood cells, and platelets. These abnormal cells can contribute to clot formation.
(2) Bone marrow dysfunction: MDS affects the bone marrow, leading to ineffective hematopoiesis. This can result in abnormal blood cell components that increase the risk of clotting.
(3) Cytokine dysregulation: In MDS, abnormal cytokine production can lead to inflammation and activation of the coagulation system, promoting clot formation.

The second ABC is individual-dependent risk factors:

(4) Age and comorbidities: MDS is more common in older adults, and age itself is a risk factor for thromboembolism. Additionally, comorbidities (such as cardiovascular disease) may further elevate the risk.
(5) Blood transfusions and chemotherapy: Some MDS patients receive blood transfusions or chemotherapy, both of which can increase the risk of thromboembolism.
(6) Coagulation disorder state: The combination of abnormal blood cells, inflammation, and other factors creates a hypercoagulable state, making clot formation more likely.

Patients with MDS should be monitored for signs of thromboembolism, and preventive measures, such as anticoagulation therapy including direct oral anticoagulants, may be considered based on individual risk factors. A consultation with a cardio-oncologist is required. And risk-oriented treatment is essential based on a specialized guideline [36].

## 7. Hemophagocytic Syndrome/Hemophagocytic Lymphohistiocytosis (HPS/HLH)

MDS-associated HPS/HLH can occur due to hyper-inflammatory conditions. Some case reports have been published in literature reviews [37,38]. One case review identified only four cases with common features [39,40], indicating that MDS can be complicated with induced-HPS/HLH through inflammation due to an external cause such as infection. Infection-induced HPS/HLH is typically reported [41].

However, the inflammatory nature of MDS has also been discussed, and HPS/HLH can be systemically initiated by MDS itself rather than as a reactive response, known as malignancy-associated HPS/HLH [42]. According to one review, malignancy cases with HPS/HLH have a poorer prognosis compared to those without HPS/HLH. Some case reports have indicated that chromosomal abnormalities associated with MDS are a pathogenic etiology [38,40]. Since genetic abnormalities and chromosomal aberrations are known in familial HLH [43], it is plausible that an associated gene controlling T cell and macrophage activation could be inducible in MDS, although the specific responsible gene has not yet been elucidated.

## 8. Hemorrhage and Hematoma Formation

Hemorrhagic adverse events and hematomas can be more frequently comorbid during chemotherapy in MDS [44]. Focusing on elderly patients and the management of thrombocytopenia and its complication, hemorrhage is reviewed [45]. Thrombocytopenia is common in MDS and accounts for 10% of deaths in MDS [2]. Thrombocytopenia in MDS appears with an increased prevalence in higher-risk IPSS categories [2]. Meanwhile, MDS presenting with isolated thrombocytopenia has a poor prognosis [46], contrary to a previous report [47]. The reason for the poor prognosis of patients with MDS presenting with isolated thrombocytopenia is unclear, but it is probably caused by the indolent disease, its cytogenetic characteristics, or the rate of leukemic transformation. All these possible causes vary arbitrarily by recruited cohort. The current study has not reached a conclusive opinion [46]. However, thrombocytopenia is maximally reflected as one point when platelet counts are less than 5.0 × 104/μL in IPSS-R risk scores [48]. The impact of thrombocytopenia in MDS should be reassessed in the future.

## 9. Leukemoid Reaction

We do not describe the details of leukemoid reactions in patients with MDS. MDS-based leukemoid reactions can sometimes be aggressive and prominent. Leukocytosis is occasionally misdiagnosed as a transformation to acute myeloid leukemia, even by certified hematologists. This reaction can be triggered by infection, and leukocytosis typically eases after recovery from the infection. We should refer to this phenomenon as a “superleukemoid reaction” arising from underlying pathogenic MDS clones.

**Figure 2:**
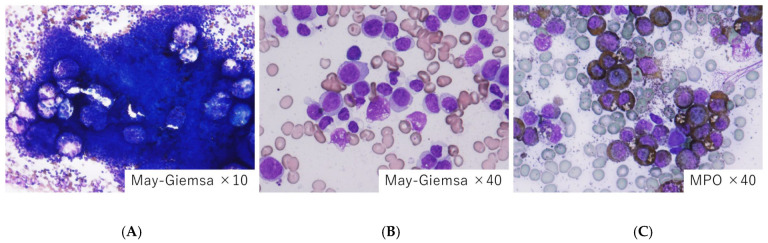
Bone marrow findings in a case of a superleukemoid reaction with MDS. (**A**) Hypercellular marrow, (**B**) increased leukocytes, and (**C**) myeloperoxidase stain. We treated an 82-year-old woman with MDS presenting with hypercellular marrow with bicytopenia (neutropenia and anemia) at onset. The patient’s peripheral blood presented an increase in leukocytes including myeloid blasts, resembling a progression to leukemia. However, a proportion of myeloid blast both in the bone marrow and peripheral blood did not increase over 20%. This leukemoid reaction recovered after the treatment of the infection. The patient was simply affected by pneumonia.

We treated an 82-year-old woman with MDS presenting with hypercellular marrow with bicytopenia (neutropenia 980/μL and anemia Hb 7.6 g/dL) at onset, who was affected by an infection (Figure 2). The laboratory data indicated acute inflammatory responses with CRP elevation (23.9 mg/dL). In the bone marrow findings, a mixture of immature myeloid cells and myeloblasts accounted for more than 20% of all nucleated cells, but this did not meet criteria of AML. My junior colleague Ms. Maki (a hematologist) misdiagnosed this case as AML and almost started induction chemotherapy after obtaining informed consent from the patient. I advised the junior colleague to prioritize treatment for infection, which resolved the patient’s leukemoid condition. The patient received the antimicrobial agent meropenem for two weeks, during which inflammatory response improved. The pneumonia was cured alongside the bicytopenia, with recovery without any cytotoxic agents. The patient is still alive with a diagnosis of low-risk MDS-MLD without specific therapy.

The presented case is not an uncommon situation. Rather than a special, rare presentation, it is low-risk MDS masquerading as AML with an inflammatory response and acute infection. MDS and coagulopathy are multifaceted and clinically significant. MDS is a clonal stem cell disorder that is characterized by ineffective hematopoiesis. This leads to cytopenias, including thrombocytopenia, which contributes directly to the risk of bleeding. This hematopoietic reaction is enhanced by cytokinemia caused by an inflammatory response, such as an infection [49]. In patients with MDS, this hematopoietic reaction leads to coagulopathy involving an excessive immune reaction and dysregulated proinflammatory cytokines [50]. This inflammatory reaction is an intrinsic property of MDS and is considered a paraneoplastic syndrome (PNS). PNSs may trigger consumptive coagulopathies or fibrinolytic processes in situations such as systemic inflammation. In these syndromes, qualitative and quantitative platelet defects in MDS can impair clot formation, even when platelet counts are not critically low. Thus, a leukemoid reaction in MDS induces coagulopathy as a secondary disease. Moreover, an intrinsic property of MDS as a paraneoplastic syndrome (PNS) also underlies the pathogenesis of coagulopathy in MDS. In the next chapter, we propose differentiating secondary and primary coagulopathies in MDS from the perspectives of clinical and molecular pathways.

## 10. Differentiate Secondary Causes from Primary Causes

**Figure 3:**
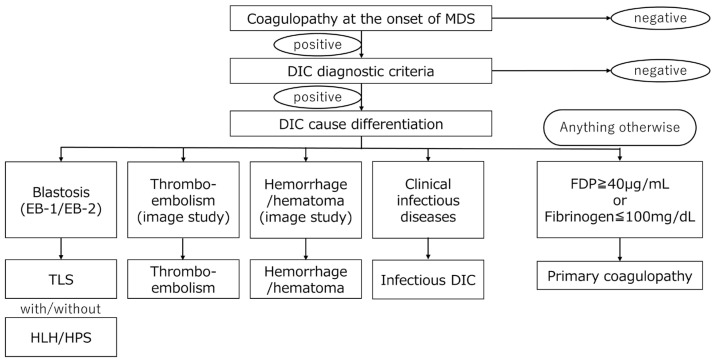
A flowchart for differentiating coagulation and fiblinolysis disorder complicated with initial MDS diagnosis. After the differentiation of the secondary cause of coagulopathy, primary coagulopathy with MDS is differentiated as paraneoplastic syndrome (PNS). (Abbreviations: DIC; disseminated intravascular coagulation, HPS/HLH; hemophagocytic syndrome/hemophagocytic lymphohistiocytosis, TLS; tumor lysis syndrome).

The clinical situation mentioned above could involve multiple factors in a single individual. This means that the reasons for coagulopathy in MDS can be multifactorial. However, we can start diagnostic examination including bone marrow testing, along with treatment for infection, DIC, and TLS. Subsequently, causes can be clearly differentiated through the clinical treatment course of patients. We propose a useful flowchart for differentiating solidified fibrinolysis observed at initial MDS diagnosis (Figure 3).

Complications of infection at the onset of MDS are common, especially in MDS with neutropenia [26]. DIC caused by infection or TLS is also common in practice but underestimated. Coagulation fibrinolysis as a PNS associated with MDS is known to take the pattern of consumptive coagulation abnormality or fibrinolytic coagulation abnormality [51]. This case report describes a unique instance of a previously unreported coagulopathy in a patient with subclinical MDS [25,26] who was unresponsive to standard hemostatic treatments. The case demonstrated that an underlying paraneoplastic coagulopathy of MDS was a potential contributor to the patient’s refractory bleeding. In such cases, clinicians can observe that the patient’s coagulopathies fluctuate depending on the course of the primary disease. Coagulopathy may improve during treatment, including chemotherapy, but disease recurrence will exacerbate it.

The corresponding coagulopathy of a disease can be manifested in its molecular pathway. The molecular pathways associated with coagulopathy and MDS have been investigated. The mTOR (mechanistic target of rapamycin) pathway has been shown to play a significant role in MDS [52]. Increased activation of the mTOR pathway is crucial for hematopoietic stem/progenitor cell proliferation and differentiation. This connection is seen in Shwachman–Diamond syndrome (SDS) [53]. The role of mTOR in platelet function and dysfunction has been studied less extensively than its role in stem cell proliferation and differentiation. However, preliminary observations regarding mTOR and platelet biology have been made, including the following: (1) mTOR signaling is upregulated upon platelet activation, and both mTORC1 and mTORC2, the two main mTOR complexes, are expressed in platelets. mTOR activity is thus essential for normal platelet activation and aggregation. (2) In addition, mTOR is involved in megakaryocyte maturation and proplatelet formation. Hyperactivation or dysregulation can lead to abnormal platelet size or function and impaired release of functional platelets. Rapamycin (sirolimus) and its analogs can impair platelet function by reducing aggregation responses and increase the bleeding risk in some transplant patients. This clinical observation supports the functional role of mTOR signaling in platelet activity.

**Figure 4:**
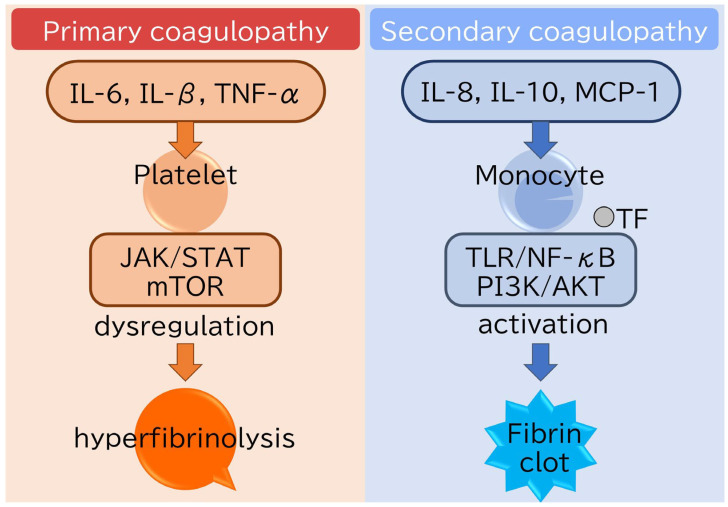
A conceptual schema of molecular mechanisms of primary and secondary coagulopathy in MDS. Primary coagulopathy in MDS is given by a genetic or intrinsic mechanism, represented by the mTOR signal pathway (left panel), while secondary coagulopathy is activated by the TLR/NF-kB pathway (right panel). (Abbreviations: MCP-1, monocyte chemotactic protein-1; NF-kB, nuclear factor kappa-light-chain-enhancer of activated B cells; mTOR, mammalian target of rapamycin; TLR, toll-like receptor).

SDS is a rare autosomal recessive disorder primarily caused by mutations in the SBDS gene, which plays a role in ribosome biogenesis and mitotic spindle stabilization. Hematological complications are central to its clinical presentation and the prognosis of SDS [54]. One clinical manifestation of SDS is increased platelet activation. This activation appears to compensate for the defective proliferation of myeloid progenitors and enhance the differentiation capacity. Similar clinical pathology is observed in some MDS cases as well. This mechanism can explain the coagulopathy in SDS and MDS as a PNS. Compared to the primary cause of coagulopathy from the mTOR pathway, secondary coagulopathy associated with MDS is caused by various complications mentioned above. The mainstay of the signaling pathway associated with secondary coagulopathy is the TLR/NF-κB pathway [55]. We provide a comparable list of molecular pathways between primary and secondary coagulopathy in Table 1 and present a demographic figure (Figure 4). In summary, MDS can present with coagulopathy as either a primary disease property or a secondary consequence of the disease. Accurate coagulopathy diagnosis and precise cause detection are essential to successfully managing patients.

## 11. Conclusions

Instead of the traditional “one-size-fits-all” model—where patients with the same diagnosis receive the same treatment—personalized medicine uses tools like laboratory data patterns, biomarker examination, and phenotypic analysis to identify what therapies are most likely to work for a specific person. For example, in cancer care, doctors might analyze a tumor’s genetic mutations to select a drug that targets those specific changes. This can lead to better outcomes, fewer side effects, and more efficient use of healthcare resources. Likewise, in the treatment of coagulopathy, discriminating the cause and detecting pathogenesis are essential to elucidate the underlying background and select the therapeutic policy.

This work contributes to the advancement of personalized medicine in MDS by proposing a structured approach to distinguish between primary and secondary causes of coagulopathy and fibrinolytic abnormalities. Recognizing that patients with MDS exhibit diverse hemostatic profiles, our diagnostic flowchart supports individualized assessment and management. By integrating clinical findings with immunophenotypic and cytokine-related insights, this approach allows clinicians to tailor interventions based on the underlying mechanism of bleeding, ultimately improving patient outcomes and avoiding unnecessary or ineffective treatments. We hope that this review will assist clinicians in their clinical practice.

## Figures and Tables

**Table 1:** Molecular pathways in coagulopathy associated with MDS.

Category	Primary Coagulopathy	Secondary Coagulopathy
Initiating mechanism	Intrinsic platelet dysfunction; dysregulated fibrinolysis linked to clonal hematopoiesis	Triggered by systemic processes like infection, DIC, TLS, HPS/HLH, and bleeding
Key cytokines	IL-6, TNF-α, and IL-1β Impact on megakaryopoiesis; clotting protein expression	IL-8, MCP-1, and IL-10 Systemic inflammation; endothelial activation
Signaling pathways	**JAK-STAT**: dysregulated in pre-MDS and CHIP **mTOR**: impairs platelet granule release	**TLR/NF-κB**: activated in infection **PI3K/AKT**: amplified in TLS **TF pathway**: endothelial/tumor cell activation
Paraneoplastic involvement	Coagulopathy as a direct PNS manifestation, independent of systemic triggers	Coagulopathy as part of broader secondary syndromes (e.g., DIC due to sepsis or bleeding)
Fibrinolytic behavior	Primary hyperfibrinolysis; abnormal plasmin generation	Secondary fibrinolysis disturbance from hepatic dysfunction; consumptive coagulopathy
Platelet function	Intrinsic thrombocytopathy: abnormal receptor expression, impaired aggregation	Quantitative platelet loss or dysfunction due to marrow suppression or peripheral consumption
Laboratory findings	Normal fibrinogen Prolonged APTT/PT Low aggregation Normal D-dimer	↓ Fibrinogen ↑ D-dimer ↑ Schistocytes due to hemolysis ↓ Platelets due to consumption
Therapeutic focus	Modulation of intrinsic pathways: mTOR, cytokines; transfusion is often insufficient	Addressing underlying disease (infection, TLS); supportive care with fresh plasma or anticoagulants if thrombotic component is present

(Abbreviations: NF-κB, nuclear factor kappa-light-chain-enhancer of activated B cells; mTOR, mammalian target of rapamycin; PI3K, phoinositide 3-kinase; TF, tissue factor; TLR, toll-like receptor). ↓ decrease, ↑ increase.

## Data Availability

No new data were created in this article.

## Abbreviations

The following abbreviations are used in this manuscript:

MDS	Myelodysplastic syndrome
MDS-MLD	Myelodysplastic syndrome with multilineage dysplasia
DIC	Disseminated intravascular coagulation
LDH	Lactate dehydrogenase
MCP-1	Monocyte chemotactic protein-1
mTOR	Mammalian target of rapamycin
NF-kB	Nuclear factor kappa-light-chain-enhancer of activated B cells
PI3K	Phoinositide 3-kinase
TF	Tissue factor
TLR	Toll-like receptor
TLS	Tumor lysis syndrome
HPS/HLH	Hemophagocytic syndrome/hemophagocytic lymphohistiocytosis
PNS	Paraneoplastic syndrome
PNSs	Paraneoplastic syndromes
SDS	Shwachman–Diamond syndrome
VEXAS	Vacuoles, E1 enzyme, X-linked, autoinflammatory, somatic
